# Novel Ultrasound-Guided Radiofrequency Ablation of the Medial Epicondylar Sensory Nerve for Recalcitrant Medial Epicondylosis: A Case-Based Technical Report

**DOI:** 10.7759/cureus.50131

**Published:** 2023-12-07

**Authors:** Devin M Jagow, Yin-Ting Chen

**Affiliations:** 1 Department of Orthopedics and Rehabilitation, Walter Reed National Military Medical Center, Bethesda, USA; 2 Department of Rehabilitation, Uniformed Services University of Health Science, Bethesda, USA

**Keywords:** chronic pain, radiofrequency ablation, epicondylitis, epicondylosis, elbow pain, ultrasonography, tendon innervation

## Abstract

Recalcitrant medial epicondylosis (ME) is a chronic tendinopathy affecting the common flexor-pronator tendon origin which causes significant pain and functional limitations. Recalcitrant ME is difficult to manage with non-surgical treatment options. The medial epicondylar sensory nerve (MEsn) is a small sensory nerve that travels within the medial intermuscular septum to innervate the osseous-tendinous structures of the medial epicondyle. In this report, we describe a novel technique for the treatment of recalcitrant ME via radiofrequency ablation (RFA) of the MEsn under ultrasound guidance. The MEsn is localized under ultrasound in the medial distal arm, just proximal to the medial epicondyle. Patients with a positive prognostic block of the MEsn subsequently underwent RFA of the MEsn. We have performed this procedure on two patients who have demonstrated improvement in pain and function for up to nearly one year after the procedure. The relief from pain and improvement in function of these patients warrants further investigation and comparative trials with respect to conventional treatment options, as MEsn RFA may be a viable treatment option for recalcitrant ME.

## Introduction

Medial epicondylitis is a common medial elbow problem affecting adults. The chronic form of medial epicondylitis is medial epicondylosis (ME), which is a tendinopathy affecting the common flexor-pronator tendon origin of the medial elbow. ME is caused by excessive repetitive stress and is exacerbated by activities involving wrist flexion. ME causes pain, and functional impairment, and reduces productivity. ME is most common in the fourth and fifth decades of life, most often occurs in the dominant arm, and has a general population incidence of 0.4% [1]. In the manual worker population, ME prevalence is estimated at 4%-5%, with annual incidence estimated at 1.5% [2].

Standard conservative treatments include occupational or physical therapy, activity modifications, orthotics, and steroid injections. Most patients with ME generally respond favorably to these treatments [3,4]. New innovative treatment options are emerging including light waves of varying wavelengths, extra-corporal shock wave therapy, platelet-rich plasma (PRP), and other orthobiologic treatments, with various reported degrees of efficacy. Specific to elbow epicondylitis, corticosteroid injection (CSI) has shown greater short-term improvement, while PRP has shown greater long-term improvement in pain and function. There is some evidence that CSI results in poorer long-term outcomes for tendinopathy [5]. PRP injections for elbow tendinopathies demonstrate improvement in pain and function, but the difference in comparison to controls has often not been consistent enough to reach consensus [6]. PRP has also shown equivocal outcomes for ME in comparison to surgery [6] and ultrasound guide percutaneous tenotomy [7] procedures.

Despite this variety of treatment options, a small percentage of ME patients will develop recalcitrant ME with continued pain and impairment. Based on a national database analysis of patients from 2007 to 2014, it’s estimated that 2% of ME patients develop recalcitrant ME, which is defined as failing standard conservative non-surgical measures [8]. The current treatment of choice for recalcitrant ME is surgical management via open, arthroscopic, or percutaneous approaches. A recent systematic review of surgical outcomes for recalcitrant ME found success rates of 63% to 100%, a complication rate of 4.3%, and a return to work/sport rate of 66.7%-100% [9]. Reported timelines for clearance to return to work and sport ranged from eight to 16 weeks in this review.

Radiofrequency ablation (RFA) is a treatment for chronic musculoskeletal pain conditions. RFA is conducted by placing a radiofrequency (RF) probe in direct contact with the target terminal sensory nerve innervating the painful structure, followed by thermocoagulation of the sensory nerve to dampen or sever the sensory connection from the painful structure. RFA treatment is well-established for the management of knee pain, facetogenic back pain, and hip pain [10,11]. Having well-defined terminal sensory nerves to the painful structure is a prerequisite for RFA. Tendons are known to receive their sensory innervation through nearby muscular, cutaneous, and peritendinous nerves [12]. However, there are relatively few reports describing the terminal sensory innervation of tendinous structures. As such, there are very limited reports regarding the use of RFA for the management of tendinopathy.

Recent investigations have led to a more in-depth understanding of elbow innervation. Dellon et al. [13] dissected 12 fresh human cadaver arms from the axilla to the medial epicondyle. They isolated a small-caliber sensory nerve within the medial intermuscular septum, which branches off from the radial nerve in the axilla alongside the branches to the medial head of the triceps. The nerve coursed alongside the ulnar nerve in the upper arm within the medial intermuscular septum distally until terminating in the periosteum of the medial humeral epicondyle, innervating the origin of the flexor-pronator muscle mass. This knowledge of the sensory innervation to the medial epicondyle lends itself as a therapeutic target for pain relief. Dellon reported successful treatment of recalcitrant ME by surgical neurectomy of the medial epicondylar sensory nerve (MEsn) in a case series of 11 patients and a single case report, with one year of follow-up demonstrating painless activity [14,15]. To our knowledge, there are no reports of non-surgical treatments targeting the MEsn. We hypothesize the application of RFA to this sensory innervation of the medial epicondyle as a viable nonsurgical treatment alternative. In this report, we describe a technique we have developed to identify the MEsn under ultrasound guidance and perform ultrasound-guided MEsn RFA for the treatment of recalcitrant ME.

## Case presentation

Two patients with recalcitrant ME were identified as candidates and underwent RFA of the MEsn after exhausting non-surgical treatment options. Patient one was a 43-year-old male with past medical history of obstructive sleep apnea, who presented with left-sided recalcitrant ME. Patient two was a 45-year-old male with a past medical history of low back pain, post-traumatic stress disorder, and traumatic brain injury, who presented with right-sided recalcitrant ME. Imaging studies of the elbow demonstrated chronic ME in both patients. MRI of the left elbow in patient one demonstrated common flexor tendinosis with a partial tear. A diagnostic ultrasound of the right elbow in patient two demonstrated a hypoechoic lesion at the common flexor tendon origin at the medial epicondyle. Both patients were recalcitrant to standard non-surgical treatment including trials of occupational therapy, oral anti-inflammatory medications, topical anti-inflammatory medications, PRP, and bracing. Numerical Pain Rating (NPR) and Upper Extremity Functional Index-15 (UEFI-15) were obtained at baseline and up to 11 months follow-up (Table 1). Both patients had a positive response to the prognostic block as described below in the procedural technique section. Both patients maintained greater than 50% pain improvement at the last follow-up and achieved significant functional improvement.

**Table 1:** Baseline and follow-up NPR and UEFI-15 scores. NPR: numerical pain rating. UEFI-15: Upper extremity functional index 15. pMCID (positive minimal clinically important difference): 6.7% [16]

Patient		Baseline	Block	8 weeks	4-6 months	9-11 months
1	NPR	7/10	2/10	2/10	2/10	4/10
	UEFI-15 (%)	39/60 (65%)		54/60 (90%)	53/60 (88.3%)	48/60 (80%)
2	NPR	8/10	0/10		2/10	3/10
	UEFI-15 (%)	50/60 (83.3%)			55/60 (91.6%)	54/60 (90%)

Scanning of the MEsn

**Figure 1:**
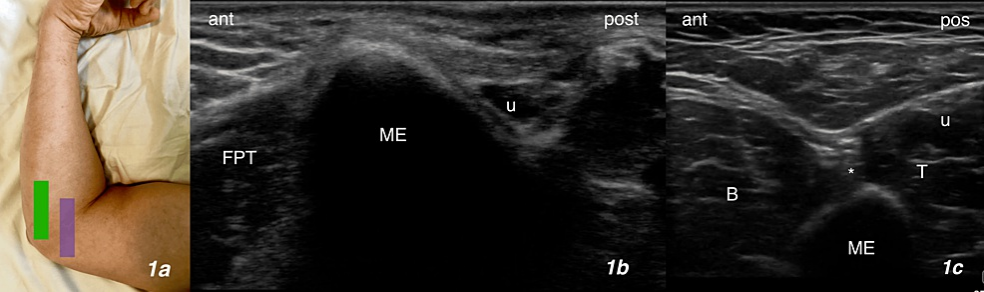
Scanning and Identification of the MEsn (a) The position of the arm during the procedure. (b) The ultrasound image at the starting position over the medial epicondyle (corresponding to the green bar in (a)). (c) Scanning slightly proximal to the medial epicondyle (corresponding to the purple bar in (a)). ant - Anterior, post - Posterior, FPT - Flexor-pronator tendon origin, ME - Medial epicondyle, B - Brachialis, (*) - MEsn. T - Triceps, u - Ulnar nerve

The patient was placed in a lateral decubitus position on the exam table, with the forearm supinated, shoulder forward flexed 90 degrees, and elbow flexed 90 degrees, a high-frequency transducer was placed over the medial epicondyle in the transverse axis to the arm (Figure 1a, green bar). The medial epicondyle is seen as a rounded bony protuberance, anterior to which is the antebrachium subcutaneous tissue and the pronator-flexor muscle tendon origin, and posterior to which is the ulnar nerve within the retrocondylar groove and the triceps (Figure 1b). Moving the transducer in short-axis slide proximally toward the shoulder (Figure 1a, purple bar), the medial epicondyle recedes away from the field of view and the intermuscular septum comes into view, dividing the brachialis in the anterior compartment and the triceps in the posterior compartment; slit of pronator teres may also be visible in the anterior compartment. The intermuscular septum is filled with adipose tissue and loose connective tissue, giving it a heterogeneic and generally hyperechoic appearance. The MEsn is seen within the intermuscular septum as a small caliber nerve, with two to three visible fascicles and minimal hyperechoic epineurium (Figure 1c). Tracing the nerve proximally to the midbrachium, the MEsn continues its course within the intermuscular septum and does not become a part of the ulnar nerve.

Procedural Technique

**Figure 2:**
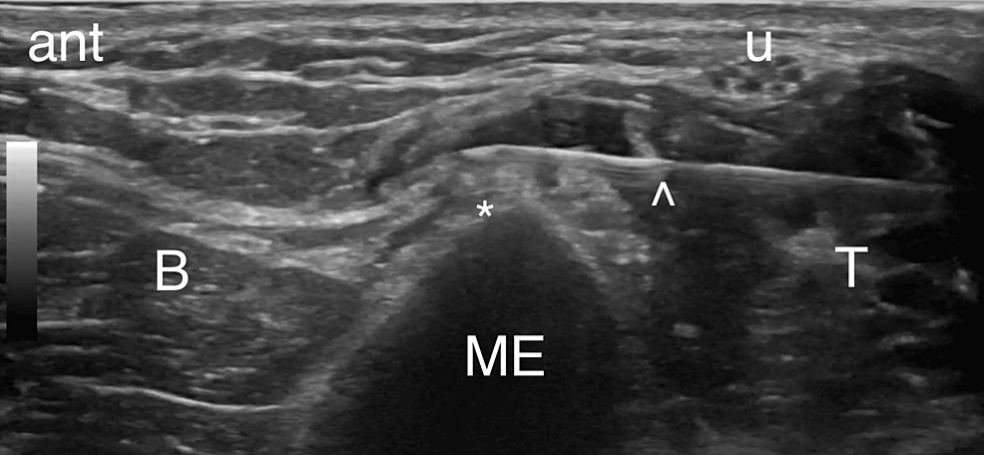
Posterior-to-anterior approach for prognostic block of the MEsn. ant - Anterior, B - Brachialis, ME - Medial epicondyle, T - Triceps, (*) - MEsn. u - Ulnar nerve, ^ - Needle.

A prognostic block was first performed in the following manner. Using the same patient positioning as described above, the medial elbow region was prepared with chlorhexidine solution and the procedure was conducted under sterile technique. A high-frequency transducer was covered with a sterile probe cover and used to visualize anatomic structures during the procedure. Local anesthesia was achieved by injecting intradermally 1% lidocaine using a 27-gauge, 1-1/4-inch needle. After the MEsn was identified using the ultrasound technique described above, a 22-gauge, 3-1/2-inch needle entered the tissue overlying the triceps posterior to the ulnar nerve and was guided in an in-plane, posterior-to-anterior approach until reaching the MEsn (Figure 2). 0.5 mL of 1% lidocaine was injected, and the patients were discharged with a pain diary. A prognostic block was considered positive if greater than 50% pain relief was achieved. 

**Figure 3:**
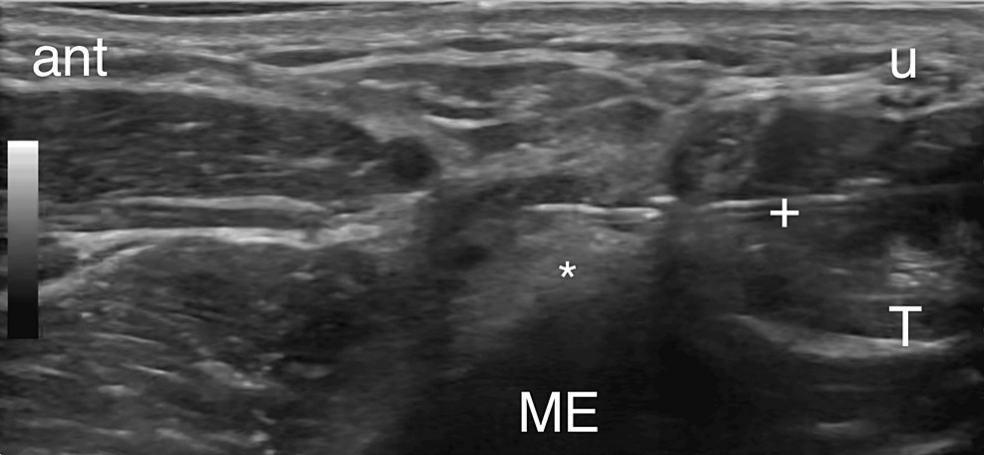
Posterior-to-anterior approach for RF probe placement and treatment of the MEsn. ant - Anterior, ME - Medial epicondyle, T - Triceps, (*) - MEsn, u - Ulnar nerve, + - RF probe.

Patients with positive prognostic block were brought back for RFA one week from the block. Using the same setup and technique as described above, a 20-gauge, 50 mm RF probe (RFP-100A RF Puncture Generator, Baylis Medical, Montreal, Canada) was positioned to maintain direct contact with its active tip with MEsn (Figure 3). Sensory testing was positive at <0.5 V at 50 Hz and motor testing was negative at 1.5 V at 2 Hz. A mixture of 1mL of 2% lidocaine was injected and allowed 90 seconds to establish pre-ablation analgesia. With direct ultrasound visualization of the RF probe in the proper position, RFA was performed at one cycle of 85°C for 90 seconds, followed by the injection of 0.5 mL of 0.50% bupivacaine and 10 mg of dexamethasone.

## Discussion

There are a number of anatomical and technical factors to consider when performing this procedure, which we detail below. Most importantly, providers must be cognizant of nearby vasculature and peripheral nerves to prevent iatrogenic injury. The ulnar nerve resides just posterior to the MEsn and superficial to the triceps. Located anterior to the MEsn is the brachial artery, cephalic vein, and the medial antebrachial cutaneous nerve (MABC) is within the subcutaneous tissue medial to the distal biceps. These anatomical factors must be considered based on the ultrasound-guided approach taken. During a posterior-to-anterior approach, the RF probe enters the tissue posterior to the ulnar nerve, and courses deep to the ulnar nerve, through the triceps muscle, in order to reach the MEsn. The ulnar nerve is well-insulated by the robust triceps muscle during the subsequent thermocoagulation treatment. During pre-treatment scanning with ultrasound probe, the ulnar nerve should be identified within the retrocondylar groove and confirm that no subluxation over the medial epicondyle occurs. The posterior-to-anterior approach requires the passage of the needle or RF probe through the triceps muscle; the increased discomfort should be tolerable with adequate local anesthesia injection of the needle track. If purchase through the triceps muscle is not desirable, a more superficial approach can be taken directly through the medial intermuscular septum, however, we caution use of this path due to the RFA probe's proximity to the ulnar nerve.

Alternatively, during an anterior-to-posterior approach, the RF probe enters the tissue in the medial antebrachial region. The ulnar nerve is furthest away from the RF probe in this approach, but the MABC is nearby in the overlying subcutaneous tissue, and can be difficult to visualize due to its small caliber. The Brachial artery and Cephalic vein must be identified and kept in the field of view during this anterior approach as well. Either approach can be performed safely so long as these anatomical structures are properly identified and remain in direct visualization under ultrasound throughout the procedure. Patient factors such as positioning on the table and upper extremity range of motion may help determine the approach to be used.

Identifying the MEsn is technically challenging. There are no studies to date describing the sonographic isolation and tracing of this nerve in its entirety. The nerve is small in caliber and can be difficult to discern from the loose connective tissue within the medial intermuscular septum. The most straightforward technique for identification is to begin scanning at the medial epicondyle and trace proximally until finding a branch of small caliber nervous tissue going into the medial intermuscular septum. We do not recommend tracing the nerve further proximally to confirm its branching from the radial nerve as described in the dissection study by Dellon (Dellon 2006) as this adds complexity without the additional benefit of risk reduction. Careful perineural hydro-dissection can help confirm the MEsn by accentuating the hyperechoic epineurium (Figure 2). As consistent with general guidance for RFA treatments, a prognostic block should be performed. A positive prognostic block predicts the success of subsequent RFA by confirming the nerve blocked is responsible for the painful region and that the degree of sensory interruption would bring clinically appreciable pain relief. Positive response in the sensory testing prior to the RFA also helps to confirm the correct nerve selection.

Proper placement of the RF probe is the key to success in any RFA procedure. For standard fluoroscopic guided RFA procedures without direct visualization of the nerve, a large ablative zone is desirable as this leads to a longer segment of the nerve ablated, which leads to a better result. Ultrasound guidance is essential in MEsn treatment given the small caliber of the nerve, and it facilitates the direct targeting and proper contact between the RF probe and the MEsn. Accidental skin burn from inadequate RF probe distance to the skin is a potential iatrogenic injury in RFA treatment. The MEsn may be a relatively superficial nerve given the low amount of adipose tissue in the medial elbow region. The risk of accidental skin burning can be reduced by maximizing the distance between the skin to the MEsn. This can be achieved by selecting the target site at the deepest portion of the tissue, which is generally just proximal to its entrance into the periosteum of the medial epicondyle. Another option is to place the RF probe directly over the MEsn and apply a compressive force downward, pushing down the MEsn to increase the distance between probe and the skin. Taking into consideration these anatomical factors discussed above will allow for the safest and most effective procedure to be performed.

Although orthobiologic treatments are a valuable therapeutic modality in musculoskeletal conditions, the relative earlier onset to pain relief is an advantage RFA has over orthobiologics. Orthobiologic treatments may require months to reach therapeutic benefit, while RFA should lead to an improved pain response within two weeks. This faster onset of pain relief may lead to earlier participation with therapeutic exercise, prevent absence from work or sport, and prevent development of chronic pain syndrome findings such as kinesiophobia and disuse. Another advantage of this procedure is the use of prognostic block. The prognostic block predicts the success of the procedure and determines whether or not to proceed with the procedure; patients with negative block will not undergo an unnecessary procedure. To date there are no clear prognostic factors for orthobiologic treatments. Also, although RFA is a destructive treatment, it is still less invasive than surgical management. The 8-16 weeks of activity restriction following surgical management may push patients away from that treatment option. RFA of the MEsn requires no post-procedure immobilization or activity restriction.

The use of RFA to treat recalcitrant tendinopathic pain is gradually progressing with the advancements in knowledge of terminal sensory nerves innervating osseous tendinous structures. One of our authors has previously described the successful management of recalcitrant greater trochanteric pain syndrome with RFA based on the anatomical understanding of the greater trochanteric sensory nerve [17,18]. Additional discoveries of the terminal sensory innervation to musculoskeletal structures may translate into more novel treatments utilizing RFA for painful musculoskeletal conditions. Alternatively, trials of pulsed-RF, chemodenervation, and peripheral nerve stimulation (PNS) may be considered for the management of these painful musculoskeletal conditions as well.

## Conclusions

This report describes the first successful treatment of ME by ultrasound-guided MEsn RFA. The success detailed in this case report warrants further discussion and exploration of terminal sensory nerve RFA as a treatment option for other tendinopathies. A trial of RFA for recalcitrant ME should be considered before surgical intervention given the lower risk of adverse effects, lower cost, earlier participation in therapy, and return to activity. As more information is gathered on the use of RFA for terminal sensory denervation, the applications of chemo-denervation and neuromodulation may also be applied to these same neural structures.
